# *Vibrio cholerae* embraces two major evolutionary traits as revealed by targeted gene sequencing

**DOI:** 10.1038/s41598-018-19995-7

**Published:** 2018-01-26

**Authors:** Kazuhisa Okada, Warawan Wongboot, Siriporn Chantaroj, Wirongrong Natakuathung, Amonrattana Roobthaisong, Watcharaporn Kamjumphol, Fumito Maruyama, Taichiro Takemura, Ichiro Nakagawa, Makoto Ohnishi, Shigeyuki Hamada

**Affiliations:** 1 Thailand-Japan Research Collaboration Center on Emerging and Re-emerging Infections (RCC-ERI), Nonthaburi, Thailand; 2 Research Institute for Microbial Diseases, Osaka University, Suita, Osaka, Japan; 3 National Institute of Health, Department of Medical Sciences, Ministry of Public Health, Nonthaburi, Thailand; 4 Department of Microbiology, Kyoto University Graduate School of Medicine, Sakyo-ku, Kyoto, Japan; 5 Vietnam Research Station, Institute of Tropical Medicine, Nagasaki University, Hanoi, Vietnam; 6 Department of Bacteriology, National Institute of Infectious Diseases, Shinjuku-ku, Tokyo, Japan

## Abstract

*Vibrio cholerae* inhabits aquatic environments worldwide and has over 200 recognized serogroups classified by O-polysaccharide specificity. Here, we report that *V*. *cholerae* selects either of two genetic traits during their evolution. Sequencing of the specific gene locus MS6_A0927 revealed that 339 of 341 strains of *V*. *cholerae* and closely related *Vibrio* species originating from 34 countries over a century carried either *metY* (*M*) (~1,269 bp) or *luxR-hchA* (*LH*) (~1,600 bp) genes, and consequently those vibrios were separated into two clusters, M (45.4%) and LH (54.6%). Only two strains contained both *M* and *LH* in the same locus. Moreover, extensive polymorphisms in those genes were detected in *M* and *LH* with 79 and 46 sequence variations, respectively. *V*. *cholerae* O1 strains isolated from cholera outbreaks worldwide, and some non-O1 strains evolving from O1 via exchange of genes encoding cell surface polysaccharides possessed *LH* alleles. Analysis of polymorphisms in the gene locus implicated a high degree of genetic diversity and identical subpopulations among the *V*. *cholerae* species.

## Introduction

*Vibrio cholerae* is a gram-negative bacterial pathogen responsible for cholera, and several million cholera cases including 21,000–143,000 deaths occur worldwide each year^1^. Serological grouping of *V*. *cholerae* has identified up to 206 O-serogroups^2^. Epidemic/pandemic cholera is typically ascribed to serogroup O1; however, in 1992, a novel serogroup O139 *V*. *cholerae* caused outbreaks in Asian countries^3^. *V*. *cholerae* carries several virulence-related genes to provoke pathogenic processes in the infected hosts. The key virulence factors of serogroups O1 and O139 include cholera toxin (CT), which is responsible for profuse watery diarrhea, and a pilus colonization factor known as toxin-coregulated pilus (TCP). Although most non-O1/non-O139 or environmental isolates of *V*. *cholerae* do not produce CT and lack the cholera toxin genes, some strains possess heat-stable enterotoxin (Stn)^4^, hemolysin (HlyA)^5,6^, repeat in toxin (RTX)^7^, Cholix toxin (ChxA)^8,9^, hemagglutinin protease (HAP)^10^, type 6 secretion system (T6SS)^11^, or type III secretion system (TTSS)^12^. However, the pathogenic mechanisms of these isolates remain to be elucidated.

High throughput sequencing facilitates the rapid and accurate identification of virulence factors of pathogenic bacteria, and can be used to identify the pathways of infectious disease transmission^13–15^. Although genomic technologies are rapidly evolving, their widespread implementation in clinical microbiology laboratories and for monitoring public health is limited owing to the need for effective semi-automated pipelines, standardized quality control and data interpretation, bioinformatics expertise, and infrastructure^16^. Relatedness and differences among *V*. *cholerae* isolates have been investigated by several molecular fingerprinting methods for a prolonged duration^17^. Pulsed field gel electrophoresis (PFGE) has been used frequently for typing of the O1 and O139 serogroups of *V*. *cholerae*^18,19^. Although PFGE is highly reproducible and its discriminatory power is sufficiently high, it is laborious, and is limited with regard to intra- and inter- laboratory comparison compared to sequence-based methods^17,20^. Multilocus sequence typing (MLST) overcomes the poor portability of traditional and older molecular typing approaches^20^. It is a technique whereby several internal control genes (loci) are sequenced, and relatedness among isolates is displayed as a dendrogram constructed using the matrix of pairwise differences between the allelic profiles. This approach provides high discriminatory power and is informative for the study of *V*. *cholerae*^21,22^. Single locus sequence typing (SLST) also has been widely used to determine the relationships in other organisms^23–25^.

We found that *V*. *cholerae* O1 genomes possess either *metY* or *hchA/luxR* on the specific gene locus MS6_A0927 on a conserved syntenic region of the chromosome II^26^. The locus was evidence that a unique O1 strain MS6 isolated from a diarrheal patient was distinguished from pandemic O1 strains and was most closely related to US Gulf Coast strains. MS6 and US Gulf Coast strains carried the *metY* gene in the locus, whereas the seventh cholera pandemic O1 strains carried *luxR-hchA* genes. In this paper, we report the prevalence, distribution, and sequence diversity of alternative genes located in MS6_A0927 among a large population of diverse *V*. *cholerae* and discuss their future evolutionary aspects.

## Results and Discussion

### *V*. *cholerae* separates into two clusters based on the locus MS6_A0927

**Figure 1:**
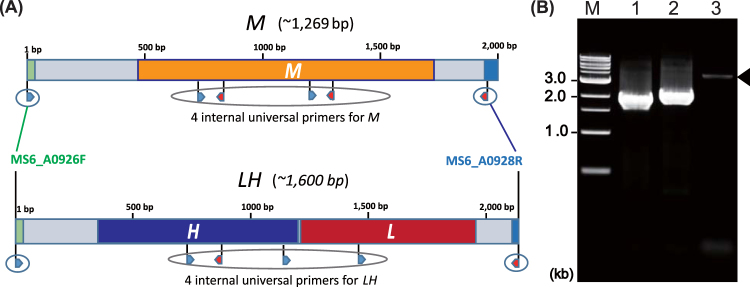
Strategy for determination of the *M and LH* sequences in the locus MS6_A0927. (**A**) Schematic diagram of *M* (upper) and *LH* (lower) regions between MS6_A0926 and MS6_A0928 and positions of sequencing primers. (**B**) PCR products obtained using the universal primers MS6_A0926 and MS6_A0928. Lane M, 1 kb DNA ladder; lane 1, *Vibrio cholerae* O1 El Tor MS6; lane 2, *V*. *cholerae* O1 El Tor N16961; lane 3, *V*. *cholerae* O35 N2_17. The expected sizes of PCR products for MS6 (*M*) and N16961 (*LH*) were 1.96 and 2.13 kb, respectively. Arrowhead indicates the amplicon that contained both sequences *M* and *LH*. All serotyped strains of non-O1 *V*. *cholerae* (n = 153) and *V*. *mimicus* (n = 2) from our bacterial stock were examined by this strategy and the results are presented in Table 1.

We investigated the distribution of the *metY* (*M*) gene and *luxR-hchA* (*LH*) genes among vibrios. First, BLAST searches were performed using *M* (1,269 bp) from *V*. *cholerae* strain MS6 and *LH* from *V*. *cholerae* strain O395 (1,600 bp) as query sequences against 186 genomes including 178 *V*. *cholerae*, 6 *V*. *mimicus*, 1 *V*. *metecus*, and 1 *V*. *parilis* obtained from the NCBI database (www.ncbi.nlm.nih.gov). All strains carried either *M* (n = 57) or *LH* (n = 128), except for strain 87395 that was revealed to carry both *M* and *LH* (Table S1). We then designed a multiplex PCR system for detection of *M*, *LH*, *toxR*, *VC2346*, *tcpA*, and *ctxAB*, and determined the sequences of *M* and *LH* in the locus MS6_A0927 of 153 strains of non-O1 *V*. *cholerae* and 2 strains of *V*. *mimicus* (Table 1). Eleven genotypes were obtained by the multiplex PCR assay, including *toxR/M* (53.5%), *toxR/LH* (30.3%), *ctxAB/tcpA/toxR/VC2346/LH* (3.2%), *ctxAB/tcpA/toxR/M* (2.6%), *ctxAB/tcpA/toxR/LH* (2.6%), *toxR/VC2346/M* (2.6%), *tcpA/toxR/M* (1.9%), *tcpA/toxR/LH* (1.3%), *tcpA/toxR/VC2346/M* (0.6%), *tcpA/toxR/VC2346/LH* (0.6%), and *M* (0.6%). Two major virulence genes, *ctxAB* and *tcpA*, were detected in 13 strains from 9 different serogroups (O8, O26, O37, O44, O48, O49, O75, O141, and O191). Moreover, the *M* gene was detected in 94 *V*. *cholerae* and 2 *V*. *mimicus* (62%, 96/155), whereas 59* V*. *cholerae* (38%, 59/155) carried *LH*. PCR using the primers MS6_A0926F and MS6_A0928R prior to sequencing of *M* or *LH* amplified target regions with expected molecular sizes, 1.96 kb or 2.13 kb, in all test strains except for *V*. *cholerae* O35 N2_17 (Fig. 1). The DNA sequences of the PCR products were determined and the existence of *M* and *LH* was confirmed. Strain N2_17 carried both *M* and *LH* in the locus, although the multiplex PCR failed to detect its *M* gene. *L* and *H* were always detected together.

**Table 1:** Characteristics of non-O1 *Vibrio cholerae* and *V*. *mimicus* as revealed by multiplex-PCR assays and *M/LH* profiling.

Serogroup	ID #	Category	Year	Location	Multiplex-PCR						Subclade	Serogroup	ID #	Category	Year	Location	Multiplex-PCR						Subclade
					*ctxAB*	*tcpA*	*toxR*	*VC2346*	*LH*	*M*							*ctxAB*	*tcpA*	*toxR*	*VC2346*	*LH*	*M*	
O2	N1	C	U	U	−	+	+	−	−	+	M64	O75	N2_31	C	1979	India	−	−	+	−	+	−	M61
O3	N2	C	U	U	−	−	+	−	+	−	LH6	O76	N50	U	U	U	−	−	+	−	+	−	LH31
O5	N3	C	1964	Philippines	−	−	+	−	−	+	M53	O77	N51	U	U	U	−	−	+	−	−	+	M47
O6	N4	C	1962	India	−	+	+	−	−	+	M30	O78	N52	U	U	U	−	−	+	−	+	−	LH12
O6	N2_2	C	U	Japan	−	−	+	−	−	+	M68	O79	N53	U	U	U	−	−	+	−	−	+	M37
O8	N2_3	C	1993	Argentina	−	−	+	−	−	+	M69	O80	N54	U	U	U	−	+	+	−	+	−	LH37
O8	N2_4	C	1962	Philippine	−	−	+	−	−	+	M58	O81	N55	U	U	U	−	−	+	−	−	+	M6
O8	N2_5	C	1994	Thailand	+	+	+	−	−	+	M50	O82	N56	U	U	U	−	−	+	−	+	−	LH4
O9	N5	C	1968	Philippines	−	−	+	−	+	−	LH41	O83	N57	U	U	U	−	−	+	−	+	−	LH10
O10	N6	C	1968	India	−	−	+	−	−	+	M70	O84	N2_32	C	1983	India	−	−	+	−	−	+	M26
O11	N7	C	1962	India	−	−	+	−	−	+	M67	O85	N58	U	U	U	−	−	+	−	+	−	LH40
O12	N8	C	1972	India	−	−	+	−	+	−	LH1	O86	N59	U	U	U	−	−	+	−	−	+	M6
O13	N9	C	1962	Philippines	−	−	+	−	−	+	M35	O87	N60	U	U	U	−	−	+	−	−	+	M51
O14	N2_6	C	U	Japan	−	−	+	−	+	−	LH1	O88	N61	U	U	U	−	−	+	−	−	+	M71
O14	N2_7	C	1964	India	−	−	+	−	+	−	LH1	O89	N62	U	U	U	−	−	+	−	−	+	M41
O14	N2_8	C	1993	Argentina	−	−	+	−	+	−	LH1	O90	N63	U	U	U	−	−	+	−	+	−	LH17
O14	N2_9	C	1993	Indonesia	−	−	+	−	+	−	LH1	O91	N64	U	U	U	−	−	+	−	−	+	M63
O15	N10	C	1979	India	−	−	+	−	−	+	M4	O92	N65	U	U	U	−	−	+	−	+	−	LH12
O16	N11	C	1971	India	−	−	+	−	+	−	LH23	O93	N66	U	U	U	−	−	+	−	−	+	M42
O17	N12	C	1968	India	−	−	+	−	−	+	M13	O94	N67	U	U	U	−	−	+	−	+	−	LH42
O18	N13	C	1964	India	−	−	+	−	+	−	LH24	O95	N68	U	U	U	−	−	+	−	−	+	M29
O19	N14	C	1968	India	−	−	+	−	+	−	LH43	O96	N69	U	U	U	−	−	+	−	−	+	M6
O20	N2_10	C	1980	USA	−	−	+	−	−	+	M23	O97	N70	U	U	U	−	−	+	−	+	−	LH34
O20	N2_11	C	1962	India	−	−	+	−	−	+	M23	O98	N2_34	C	1993	Indonesia	−	−	+	−	+	−	LH1
O21	N15	C	1968	India	−	−	+	−	+	−	LH24	O98	N2_35	C	1976	India	−	−	+	−	−	+	M57
O23	N16	C	1971	India	−	−	−	−	−	+	M18	O99	N71	U	U	U	−	−	+	−	−	+	M72
O24	N17	C	1962	Philippines	−	−	+	−	−	+	M34	O100	N72	U	U	U	−	−	+	−	+	−	LH1
O25	N18	C	1962	India	−	−	+	−	+	−	LH30	O102	N74	U	U	U	−	−	+	−	+	−	LH24
O26	N2_12	C	1991	Japan	+	+	+	+	+	−	LH38	O103	N75	U	U	U	−	−	+	−	+	−	LH22
O26	N2_14	C	1993	Sri Lanka	+	+	+	+	+	−	LH38	O104	N76	U	U	U	−	−	+	−	−	+	M45
O26	N2_15	C	1994	Brazil	+	+	+	+	+	−	LH38	O105	N77	U	U	U	−	+	+	−	−	+	M44
O26	N2_16	C	1996	Japan	+	+	+	+	+	−	LH38	O106	N78	U	U	U	−	−	+	−	−	+	M43
O27	N19	C	1962	Philippines	−	−	+	−	−	+	M65	O107	N79	U	U	U	−	−	+	−	+	−	LH4
O28	N20	C	1962	Philippines	−	−	+	−	−	+	M46	O108	N80	U	U	U	−	−	+	−	+	−	LH4
O30	N21 (VM)	C	1962	Philippines	−	−	+	−	−	+	M16	O109	N81	U	U	U	−	−	+	−	+	−	LH28
												O110	N82	U	U	U	−	−	+	−	+	−	LH36
O32	N22 (VM)	C	1968	India	−	−	+	−	−	+	M75	O111	N83	U	U	U	−	−	+	−	+	−	LH4
												O113	N84	U	U	U	−	−	+	−	+	−	LH46
O34	N23	C	1968	India	−	−	+	−	+	−	LH29	O114	N85	U	U	U	−	−	+	+	−	+	M77
O35	N24	C	1969	India	−	−	+	−	−	+	M37	O115	N86	U	U	U	−	+	+	+	−	+	M6
O35	N2_17	C	1994	Korea	−	−	+	−	+	+	LH19&M5	O116	N87	U	U	U	−	−	+	−	−	+	M74
										a		O117	N88	U	U	U	−	−	+	+	−	+	M76
O36	N25	C	1969	Philippines	−	−	+	−	−	+	M32	O118	N89	U	U	U	−	−	+	−	−	+	M15
O37	N26	C	1969	India	+	+	+	−	+	−	LH1	O119	N90	U	U	U	−	−	+	−	−	+	M51
O37	N2_19	E	1996	Korea	+	+	+	−	+	−	LH1	O120	N91	U	U	U	−	−	+	−	+	−	LH27
O39	N2_20	E	1994	Korea	−	−	+	−	+	−	LH1	O121	N92	U	U	U	−	−	+	−	−	+	M54
O39	N2_21	C	1968	India	−	−	+	−	+	−	LH33	O122	N93	U	U	U	−	−	+	−	+	−	LH28
O40	N27	C	1972	India	−	−	+	−	−	+	M26	O123	N94	U	U	U	−	−	+	−	−	+	M30
O42	N28	C	1973	India	−	−	+	−	−	+	M4	O124	N95	U	U	U	−	−	+	−	−	+	M26
O43	N29	C	1973	India	−	−	+	−	+	−	LH20	O125	N96	U	U	U	−	−	+	−	−	+	M4
O44	N2_22	C	1994	Thailand	+	+	+	+	+	−	LH32	O126	N97	U	U	U	−	−	+	−	−	+	M31
O44	N2_23	C	1973	India	−	−	+	−	−	+	M66	O127	N98	U	U	U	−	−	+	−	+	−	LH12
O45	N30	C	1973	India	−	−	+	−	−	+	M38	O128	N99	U	U	U	−	−	+	−	+	−	LH39
O46	N31	C	1973	India	−	−	+	−	−	+	M18	O129	N100	U	U	U	−	−	+	−	−	+	M26
O47	N32	C	1973	India	−	−	+	−	−	+	M4	O130	N101	U	U	U	−	−	+	−	−	+	M49
O48	N33	C	1973	India	−	−	+	−	−	+	M58	O131	N102	U	U	U	−	−	+	−	−	+	M60
O48	N2_24	E	1988	Taiwan	+	+	+	−	+	−	LH18	O132	N103	U	U	U	−	−	+	−	−	+	M4
O49	N2_25	C	1994	Thailand	+	+	+	−	+	−	LH45	O133	N104	U	U	U	−	−	+	−	−	+	M4
O49	N2_26	C	1974	India	−	−	+	−	−	+	M49	O134	N105	U	U	U	−	−	+	−	−	+	M6
O51	N34	C	1973	India	−	−	+	−	−	+	M4	O135	N106	U	U	U	−	−	+	+	−	+	M78
O52	N35	C	1973	India	−	−	+	−	−	+	M79	O136	N107	U	U	U	−	−	+	−	+	−	LH44
O54	N36	C	1974	India	−	−	+	−	−	+	M55	O137	N108	U	U	U	−	−	+	−	−	+	M36
O57	N37	C	1976	Denmark	−	−	+	−	−	+	M27	O139	N2_38	U	1993	Sri Lanka	−	−	+	−	−	+	M28
O58	N38	C	1974	India	−	−	+	−	+	−	LH47	O139	N2_39	U	1993	Pakistan	−	−	+	−	−	+	M28
O59	N39	C	1974	India	−	−	+	−	−	+	M33	O141	N2_40	C	1993	India	+	+	+	−	−	+	M14
O60	N40	C	1975	India	−	−	+	−	+	−	LH25	O141	N2_41	E	1995	Brazil	−	−	+	−	−	+	M5
O61	N41	C	1974	India	−	−	+	−	−	+	M4	O142	N109	U	U	U	−	−	+	−	−	+	M41
O64	N42	C	1975	India	−	−	+	−	−	+	M30	O144	N2_42	E	1994	Denmark	−	−	+	−	−	+	M46
O65	N43	C	1975	India	−	+	+	−	+	−	LH2	O144	N2_43	C	1993	India	−	−	+	−	−	+	M59
O66	N2_27	E	1993	Argentina	−	−	+	−	−	+	M56	O145	N111	U	U	U	−	−	+	−	−	+	M62
O66	N2_28	C	1975	India	−	−	+	−	+	−	LH16	O146	N112	U	U	U	−	−	+	−	−	+	M41
O67	N44	C	1979	India	−	−	+	−	+	−	LH26	O147	N113	U	U	U	−	−	+	−	−	+	M41
O68	N45	E	1978	Japan	−	−	+	−	−	+	M11	O148	N114	U	U	U	−	−	+	−	−	+	M39
O69	N46	C	1979	India	−	−	+	−	+	−	LH21	O149	N2_44	E	1994	Germany	−	−	+	−	+	−	LH35
O69	N2_29	E	1994	Denmark	−	−	+	−	−	+	M52	O149	N2_45	C	1993	Indonesia	−	−	+	−	−	+	M6
O71	N47	U	U	U	−	−	+	+	−	+	M73	O150	N115	U	U	U	−	−	+	−	−	+	M40
O72	N48	U	U	U	−	−	+	−	−	+	M11	O151	N116	U	U	U	−	−	+	−	−	+	M6
O74	N49	U	U	U	−	+	+	+	+	−	LH34	O152	N117	U	U	U	−	−	+	−	−	+	M4
O75	N2_30	C	2006	USA	+	+	+	−	−	+	M44	O191	N2_46	C	1996	Japan	+	+	+	−	−	+	M48

Abbreviations: C, human isolate; E, environmental isolate; U, unknown; VM, *V*. *mimicus*.

a, Only *LH* was detected by the multiplex PCR assay but *M* was determined by targeted sequencing.

**Figure 2:**
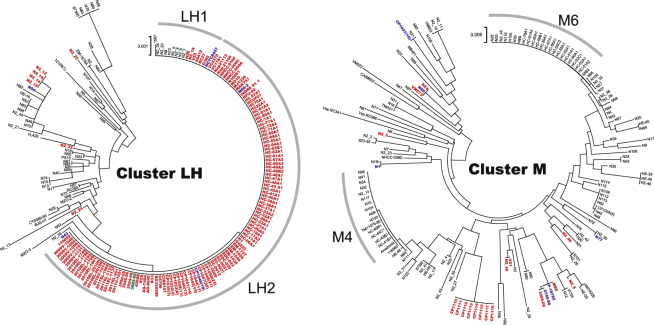
*Vibrio cholerae* organisms were classified into two clusters, M and LH. Dendrograms were constructed based on the genes *M* or *LH* from 341 *Vibrio* strains by the neighbor-joining method using MEGA v.6.0. Scale bars indicate nucleotide substitutions per site. Color coding is based on the presence of genes for cholera toxin (*ctxAB*) and toxin-coregulated pilus (*tcpA*): red, *ctxAB*+, *tcpA*+; blue, *ctxAB*−, *tcpA*+; green, *ctxAB*+, *tcpA*−; black, *ctxAB*−, *tcpA*−. The *M* and *LH* genes exhibited 79 and 46 sequence variations, respectively. The predominant subclades of *LH* and *M* were LH1, LH2, M4, and M6 in the order of description.

Overall, 339 of 341 strains of vibrios, mostly *V*. *cholerae*, carried either *M* or *LH* in the locus MS6_A0927, and consequently those vibrios were separated into two clades: M (45.4%) and LH (54.6%) (Fig. 2). The remaining two strains of *V*. *cholerae* carried both *M* and *LH* in the same locus; thus, all *V*. *cholerae* strains carried either *M* or *LH*, or both. The *M/LH* sequence profiling further classified the 341 strains of vibrios into 127 subclades.

### Evolutionary selection of *M* or *LH* in *V*. *cholerae*

We detected *LH* only in *V. cholerae* and not in other vibrios. In contrast, *M* was present in strains of *V. cholerae, V. mimicus, V. parilis, V. metecus, V. splendidus, V. cyclitrophicus, V. tasmaniensis, V. tubiashii*, and *V. jasicida*, but not in *V. fluvialis, V. furnissii, V. parahaemolyticus, V. vulnificus,* and *V. anguillarum* in publicly available database. The nucleotide sequence of *M* showed higher similarity within a single species than between different species (Fig. S1). Exceptionally, *M* from *V. cholerae* CP1037(10) showed higher similarity to that from in *V. mimicus* than to those of most *V. cholerae. V. mimicus, V. parilis,* and *V. metecus* carry *M* on the MS6_A0927 in chromosome II, similarly to *V. cholerae,* whereas *V. splendidus, V. tasmaniensis,* and *V. cyclitrophicus* carry *M* near the homologue of the glutamine-fructose-6-phosphate transaminase gene (MS6_0339), which is located on chromosome I. The latter three species and *V. tubiashii* and *V. jasicida* harbored homologous of MS6_A0926 and MS6_A0928 at various distances (0.2 kb to 10 kb) from the two loci. *V. fluvialis* and *V. furnissii,* which are more closely related to* V. cholerae* and *V. mimicus*^27^, carried homologues of the aldehyde dehydrogenase (MS6_1585) and sigma 54-dependent transcriptional regulator (MS6_1586) genes between the two loci, although they did not carry *M* and *LH*. The alternative selection of *M/LH* at MS6_A0927 would have occurred in ancestral populations of *V. cholerae*.

**Figure 3:**
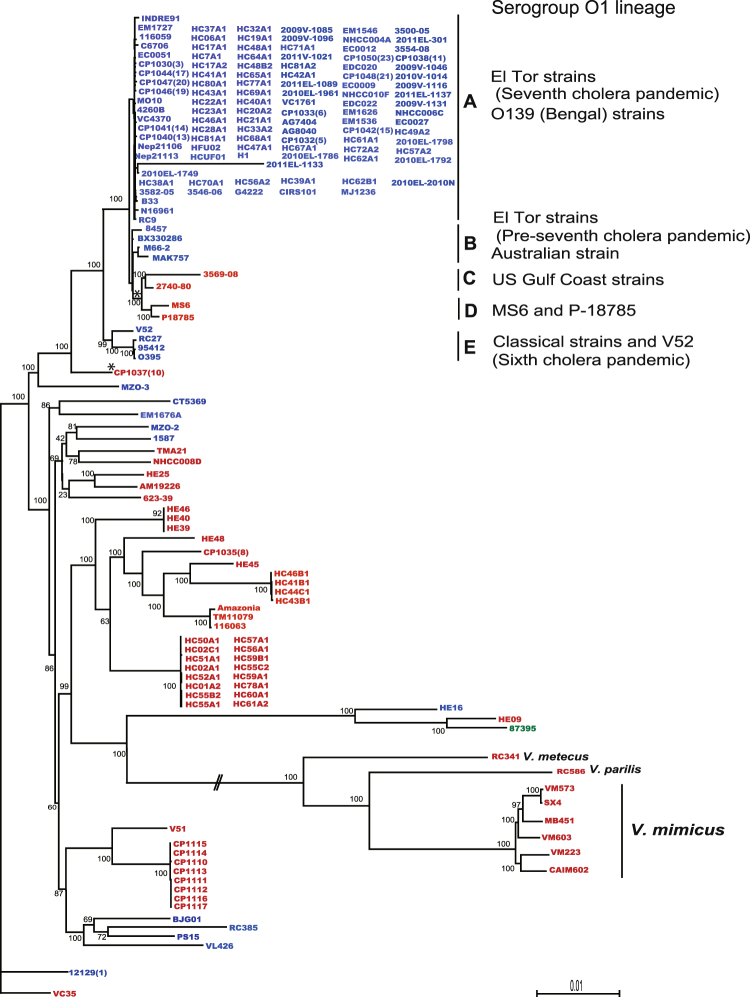
Phylogenetic relationships among *Vibrio cholerae* and other *Vibrio* spp. and distribution of genes *M* and *LH*. A maximum likelihood tree showed phylogenetic relationships among 178 strains of *V*. *cholerae*, 6 *V*. *mimicus*, 1 *V*. *metecus*, and 1 *V*. *parilis*. Color coding is based on the presence of *M*: red; *LH*: blue; and *M* and *LH*: green. Bootstrap supports (%) are indicated at branching points. Branch lengths are proportional to sequence differences. Pathogenic O1 strains were classified into five phylogenic groups, A to E. Asterisk indicates a possible recombination event for the *M* gene through horizontal gene transfer.

The distributions of *M* and *LH* in the strains of *V*. *cholerae* were generally associated with genome-based phylogeny (Fig. 3). The *V*. *cholerae* O1 lineage carried *LH*, except for the four strains in phylogenetic groups C and D. All 112 strains of groups A and B except for CP1046 exhibited subclade LH2, and the four strains of the group E showed LH1. The difference in these two subclades, LH1 and LH2, was ascribed to the absence of thymine in the 8-bp poly-T region of the *H* gene, which caused a frameshift to generate a modified protein that was shorter by 83 amino acids. The four strains in groups C and D showed subclades M2 and M1, respectively. Subclade M1 was represented by a Thai strain, MS6. This strain was very similar to the Russian strain P-18785^28^. The US gulf coast strains 2740–80 and 3569-08 were designated M2, which differed from M1 by one nucleotide. The sequences of the neighboring genes *kbl* (MS6_A0926) and *lysR* (MS6_A0928) were mostly identical among the strains of groups AB and E, but they were different from those in groups C and D, corresponding to the subclustering results for the targeted gene sequence of MS6_A0927 (Table 2). *LH* was likely replaced with *M* in the strains of groups C and D after they diverged from a common ancestor of classical and El Tor-biotype organisms.

**Table 2:** Sequence variations among the 186 vibrios as revealed by the *M/LH* sequence profiling and other 9 single locus sequence typing assays.

Strain(s)	MS6_A0927	A0926	A0928	Genes used for MLST							Group
	Subclade	*kbl*	*lysR*	*adk*	*gyrB*	*mdh*	*metE*	*pntA*	*purM*	*pyrC*	
N16961/others (n = 97)	LH2	1^a^	1	1(7)	1(11)	1(4)	1(37)	1(12)	1(1)	1(20)	A^b^
EDC−022	LH2	2	1	1(7)	1(11)	1(4)	1(37)	1(12)	1(1)	1(20)	
EM-1536	LH2	2	1	1(7)	1(11)	1(4)	1(37)	1(12)	1(1)	1(20)	
EM_1626	LH2	2	1	1(7)	1(11)	1(4)	1(37)	1(12)	1(1)	1(20)	
VC1761	LH2	2	1	1(7)	1(11)	1(4)	1(37)	1(12)	1(1)	1(20)	
CP1042(15)	LH2	2	1	1(7)	1(11)	1(4)	1(37)	1(12)	1(1)	1(20)	
2011EL-1133	LH2	***S***	1	1(7)	1(11)	1(4)	***S***(37)	***S***(NA)	***S***(NA)	***S***(NA)	
2010EL-1749	LH2	1	1	1(7)	1(11)	1(4)	NA(NA)	1(12)	1(1)	1(20)	
INDRE_91_1	LH2	1	1	1(7)	1(11)	1(4)	1(37)	1(12)	1(1)	***S***(NA)	
CP1041(14)	LH2	1	1	1(7)	1(11)	1(4)	2(206)	1(12)	1(1)	1(20)	
CP1040(13)	LH2	1	1	1(7)	1(11)	1(4)	2(206)	1(12)	1(1)	1(20)	
CP1046	***LH3***	1	1	1(7)	1(11)	1(4)	1(37)	1(12)	1(1)	1(20)	
8457	LH2	1	1	1(7)	1(11)	1(4)	1(37)	1(12)	1(1)	2(38)	B
M66-2	LH2	1	2	1(7)	1(11)	1(4)	1(37)	1(12)	1(1)	2(38)	
MAK757	LH2	1	2	1(7)	1(11)	1(4)	1(37)	1(12)	***S***(NA)	2(38)	
BX330286	LH2	1	1	1(7)	1(11)	1(4)	1(37)	1(12)	1(1)	***S***(37)	
MS6	M1	***S***	3	1(7)	2(2)	1(4)	1(37)	1(12)	1(1)	2(38)	C
P18785	M1	3	3	1(7)	2(2)	1(4)	1(37)	1(12)	1(1)	2(38)	
3569-08	M2	3	3	1(7)	***S***(NA)	1(4)	1(37)	1(12)	2(NA)	2(38)	D
2740-80	M2	3	3	1(7)	2(2)	1(4)	1(37)	1(12)	1(1)	2(38)	
O395	LH1	1	1	1(7)	1(11)	1(4)	3(9)	1(12)	1(1)	2(38)	E
RC27	LH1	1	1	1(7)	1(11)	1(4)	3(9)	1(12)	1(1)	2(38)	
95412	LH1	1	1	1(7)	1(11)	1(4)	3(9)	1(12)	1(1)	2(38)	
V52	LH1	1	1	1(7)	1(11)	1(4)	***S***(17)	***S***(23)	1(1)	***S***(35)	
HE-46/others (n = 3)	M7	4	4	2(19)	3(1)	2(14)	4(66)	2(39)	3(1)	3(45)	Others
HE-48	***M19***	5	***S***	***S***(45)	4(46)	3(3)	***S***(new)	***S***(95)	4(1)	***S***(123)	
CP-1035(8)	***M11***	***S***	***S***	3(26)	5(5)	***S***(2)	5(50)	3(30)	***S***(18)	3(45)	
HE-45	***M15***	5	***S***	4(1)	5(5)	4(14)	5(50)	4(31)	5(14)	***S***(12)	
HC-41B1	M4	5	5	3(26)	6(5)	5(new)	5(50)	4(31)	5(14)	3(45)	
HC-43B1	M4	5	5	3(26)	6(5)	5(new)	5(50)	4(31)	5(14)	3(45)	
HC-44C1	M4	5	5	3(26)	6(5)	5(new)	5(50)	4(31)	5(14)	3(45)	
HC-46B1	M4	5	5	3(26)	6(5)	5(new)	5(50)	4(31)	5(14)	3(45)	
116063	M4	5	5	3(26)	6(5)	4(14)	6(54)	5(28)	5(14)	3(45)	
TM11079-80	M4	5	5	3(26)	6(5)	4(14)	6(54)	5(28)	5(14)	3(45)	
Amazonia	M4	5	5	3(26)	6(5)	4(14)	6(54)	5(28)	5(14)	3(45)	
HC50A1/others (n = 16)	M6	6	6	5(***S***)	7(36)	2(14)	7(40)	3(30)	5(14)	4(4)	
HE_16	***LH12***	***S***	7	6(23)	8(34)	6(26)	***S***(78)	***S***(36)	6(10)	5(29)	
HE_09	***M5***	***S***	7	6(23)	4(46)	6(26)	***S***(32)	6(37)	6(10)	5(29)	
87395	***LH4M5***	***S***	***S***	6(23)	8(34)	6(26)	***S***(57)	6(37)	6(10)	***S***(25)	
SX_4	M8	7	8	7(32)	9(21)	***S***(38)	8(26)	7(46)	7(25)	6(34)	
VM573	M8	7	8	7(32)	9(21)	***S***(38)	8(26)	7(46)	7(25)	6(34)	
CAIM602	***M20***	***S***	***S***	***S***(31)	***S***(18)	***S***(41)	***S***(52)	8(40)	***S***(23)	***S***(33)	
VM223	***M22***	***S***	***S***	***S***(30)	***S***(16)	***S***(36)	***S***(62)	8(40)	***S***(24)	***S***(30)	
MB-451	***M21***	***S***	***S***	***S***(31)	***S***(22)	***S***(40)	***S***(24)	***S***(41)	***S***(28)	***S***(17)	
VM603	***M23***	***S***	***S***	***S***(35)	***S***(19)	***S***(37)	***S***(25)	***S***(45)	***S***(26)	***S***(32)	
V51	***M14***	***S***	***S***	8(25)	***S***(5)	7(22)	9(16)	9(21)	8(8)	***S***(11)	
CP1110/others (n = 8)	M3	8	9	8(25)	11(***S***)	7(22)	9(16)	9(21)	9(new)	7(new)	
MZO-3	***LH9***	***S***	***S***	***S***(4)	***S***(23)	***S***(28)	***S***(13)	10(25)	4(1)	***S***(9)	
CT 5369-93	***LH15***	***S***	***S***	NA(NA)	NA(NA)	***S***(30)	***S***(59)	11(3)	***S***(1)	3(45)	
EM-1676A	***LH6***	***S***	***S***	4(1)	***S***(90)	***S***(new)	***S***(new)	***S***(59)	3(1)	***S***(new)	
MZO-2	***LH8***	***S***	***S***	9(2)	***S***(23)	8(15)	***S***(42)	12(18)	***S***(13)	***S***(10)	
1587	***LH7***	1	1	***S***(NA)	***S***(6)	***S***(29)	***S***(2)	10(25)	10(1)	***S***(3)	
TMA21	***M18***	***S***	***S***	***S***(9)	***S***(25)	3(3)	***S***(41)	***S***(6)	10(1)	8(5)	
NHCC-008D	***M12***	***S***	***S***	***S***(new)	***S***(90)	***S***(46)	10(19)	***S***(18)	***S***(1)	***S***(new)	Others
HE-25	***M10***	9	10	9(2)	5(5)	***S***(44)	10(19)	13(6)	1(1)	***S***(51)	
AM19226	***M9***	9	10	9(2)	5(5)	***S***(13)	***S***(19)	13(6)	***S***(6)	8(5)	
623-39	***M17***	***S***	***S***	***S***(20)	***S***(41)	***S***(5)	***S***(3)	12(18)	***S***(2)	***S***(4)	
CP1037(10)	***M16***	***S***	***S***	1(7)	***S***(57)	8(15)	***S***(96)	***S***(new)	10(1)	***S***(75)	
BJG-01	***LH11***	***S***	***S***	***S***(13)	***S***(30)	2(14)	***S***(44)	11(3)	3(1)	***S***(39)	
RC385	***LH10***	***S***	***S***	***S***(2)	NA(NA)	***S***(18)	***S***(45)	***S***(4)	8(8)	***S***(48)	
PS15	***LH13***	***S***	***S***	***S***(new)	***S***(5)	***S***(14)	***S***(130)	***S***(66)	***S***(new)	***S***(new)	
VL426	***LH14***	***S***	***S***	***S***(15)	***S***(29)	***S***(16)	***S***(18)	***S***(10)	10(1)	***S***(22)	
12129(1)	***LH5***	***S***	***S***	***S***(14)	11(31)	***S***(20)	***S***(39)	***S***(16)	1(1)	9(13)	
VC35	***M13***	***S***	***S***	***S***(56)	11(31)	***S***(56)	4(66)	***S***(68)	***S***(48)	9(13)	
RC341	***M24***	***S***	***S***	***S***(28)	***S***(new)	***S***(31)	***S***(14)	***S***(27)	***S***(22)	***S***(8)	
RC586	***M25***	***S***	***S***	***S***(29)	***S***(35)	***S***(new)	***S***(51)	***S***(new)	***S***(32)	***S***(21)	
Total											
NA	0	0	1	1 (2)	2 (3)	0 (0)	1 (1)	0 (1)	0 (3)	0 (2)	
variations	40	36	35	27(24)	30(24)	30(27)	36(34)	30(27)	27(18)	35(32)	

^a^Sequence types in each gene. In italics and bold-face, singleton sequence type; NA, not available. Numbers in parentheses after the names of genes used for MLST indicate the allele number based on the profile definitions in the *Vibrio cholerae* MLST databases. ^b^Group assigned in Fig. 3.

We found a similar gene arrangement in the loci between MS6_A0926 and MS6_A0928 containing *M* and *LH* in the two strains N2_17 and 87395 (Fig. S2). Strain 87395 was phylogenetically closely related with HE-09, which exhibited M5 in common. In addition, the DNA sequence of *LH* was clearly different from those in *V*. *cholerae*, except for the four strains N56, N79, N80, and N83 of subclade LH4.

Based on these observations, *V*. *cholerae* commonly carried a single copy of the *M* and/or *LH* genes in the specific locus. It is likely that *V*. *cholerae* originally carried the *M* gene; however, horizontal gene transfer led to the emergence of *V*. *cholerae* carrying *LH* in ancient times. In some subclades of *V*. *cholerae*, such as those in groups C and D and CP1037(10), gene replacement with *M* occurred in succession. As observed in strains 87395 and N2.17, an incomplete choice between the two alternatives of *M* and *LH* could occur, resulting in incidental possession of both *M* and *LH* in these strains. The alternative choice of *M* or *LH* might be of benefit to the survival of *V*. *cholerae* in different environmental conditions. Very recently, Das *et al*. (29) reported that the product of *H* in *V*. *cholerae* O1 classical biotype strain O395 was endowed with molecular chaperone, aminopeptidase, and robust methylglyoxalase activities. The functional roles of these genes in *V*. *cholerae* are now under investigation.

### Genetic diversity and population structure of *V*. *cholerae* revealed by *M/LH* sequence profiling

The above-described data show that *V*. *cholerae* have maintained *M or LH* in MS6_A0927 on their genomes and indicate that the subclades corresponding well to clusters generated from a genome-wide phylogenetic analysis. Therefore, we considered the advantages and limitations of using our targeted gene sequencing for *V*. *cholerae* investigations. The *M/LH* sequence profiling exhibited the highest discrimination index (D = 0.63) as compared with those of the nine SLST analyses that targeted the two gene loci *kbl* and *lysR* and seven housekeeping genes *adk*, *gyrB*, *mdh*, *metE*, *pntA*, *purM*, and *pyrC* (Table 2). The *kbl* and *lsyR* genes encode 2-amino-3-ketobutyrate coenzyme A ligase and the LysR family of transcriptional regulators, respectively. *M/LH* sequence profiling differentiated between the four groups, i.e., A/B, C, D, and E, whereas the 9 other SLSTs failed to differentiate these groups. The conventional MLST analysis based on the seven loci was able to differentiate between groups A and B, but not between C and D. The ability to distinguish C from D was critical to trace the pathogen MS6 back to its likely origin in China or vice versa. Recently, sporadic cholera outbreaks caused by US Gulf-like *V*. *cholerae* O1 (non-7th pandemic clone) occurred in Zhejiang province, China^29^. MLST analysis separated 13 Zhejiang strains into 3 sequence types—ST75, ST169, and ST170—and of them, 10 strains with ST75 sequence type were identical to US Gulf coast strains, while *M/LH* sequence profiling showed that all the 13 strains belonged to group D (subclade M1), differentiating them from US Gulf coast strains (M2). The results of *M/LH* sequence profiling corresponded well with those of the clusters deduced by their whole genome sequence-based phylogenetic analysis, and consequently, it demonstrated higher epidemiological relevance than MLST did.

Vibrios with *M* or *LH* were isolated from clinical and environmental sources. Subclades LH1, LH2, M4, and M6 were dominantly found in test strains (Fig. 2). Among them, LH2 mostly comprised the seventh cholera pandemic El Tor and toxigenic O139 strains, whereas the classical type of *V*. *cholerae*, the major player in the sixth cholera pandemic, belonged to subclade LH1. Interestingly, there have been no reports on the toxigenic and non-toxigenic O1 strains in group C and D that replaced *LH* with *M* causing epidemics and severe diarrhea (Fig. 3)^28–32^. Furthermore, all 20 clinical strains of non-O1/non-O139 *V*. *cholerae* obtained in the Haiti cholera epidemic belonged to two subclades M4 and M6 (Table S1), which corresponded to clusters HC-1 and −2 as indicated by the comparative genomic analysis of Hassan *et al*. (29). The three environmental strains from Haiti from 2010 were in subclade M7. In addition, eight strains of serogroup O75 CP1110-CP1117 from an oyster-borne cholera outbreak in Florida^33^ belonged to subclade M3.

In this study, 130 strains including *V*. *cholerae* O1 and O139 were separated into 13 subclades. Subclade LH2 contained current pandemic/epidemic clone and normally possessed the genes *ctxAB*/*tcpA*/*toxR/VC234*6/*LH*, which were targeted by our multiplex PCR. Most of the non-toxigenic *V*. *cholerae* of O1 serogroup, such as strains 12129(1) and TM11079-80, belonged to different subclades, and phylogenetic lineages from the toxigenic O1 strains and their O1 antigen phenotype probably arose from horizontal gene exchange in the evolution of *V*. *cholerae*^13^. The alterations in the cell surface antigens of *V*. *cholerae* can lead to new epidemics/pandemics, especially in populations without adequate immunity against the serogroup. *V*. *cholerae* O139 Bengal evolved from a *V*. *cholerae* O1 El Tor strain by exchange of genes encoding cell surface polysaccharides^34,35^, and cholera caused by *V*. *cholerae* O139 Bengal has rapidly spread in southeast Asian countries following its initial isolation in Madras, India, in 1992. A high incidence of cholera has been observed in both adults and children in the areas where cholera is endemic^36,37^.

In conclusion, our targeted gene sequencing of MS6_A0927 revealed divergent genetic traits among *V*. *cholerae* species.

## Methods

### Bacterial strains and genomes

This study analyzed 341 genomes consisting of i) O1/O139 (n = 128), non-O1/non-O139 (n = 48), and unknown serogroups (n = 2) of *V*. *cholerae* strains; *V*. *mimicus* (n = 6); *V*. *metecus* (n = 1); and *V*. *parilis* (n = 1) deposited in GenBank; and ii) serotyped strains of non-O1 *V*. *cholerae* (n = 153) and *V*. *mimicus* (n = 2). Detailed information on the genomes/strains used in this study is presented in Table S1 and Table 1.

### Multiplex PCR method

In total, 186 genomes of *Vibrio* species (Table S1) were referenced for the in-house design of the multiplex PCR method and targeted gene sequencing. Primers for the six target genes *toxR*, *ctxAB*, *luxR-hchA* (*LH*), *metY* (*M*), *VC2346*^38,39^, and *tcpA* were designed to target each consensus region. The multiplex PCR assay for detection of those genes was performed in a 25-µl reaction mixture containing 0.2 mM dNTPs, 1 × Ex Taq buffer, 2 mM MgCl_2_, each primer at 1 µM, 0.75 U of Ex Taq DNA polymerase (Takara Bio, Otsu, Japan), and 100 ng of genomic DNA extracted using the NucleoSpin Tissue kit (Macherey-Nagel, Düren, Germany). Primer sequences are shown in Table S2. Thermal cycling conditions were as follows: 94 °C for 30 s and 30 cycles of 94 °C for 30 s, 59 °C for 1 min, and 72 °C for 1 min. Amplicons were separated by 2% agarose gel electrophoresis and bands were visualized under ultraviolet transillumination after staining of the gel with ethidium bromide (Fig. S3).

### Targeted gene sequencing

PCR amplification of the locus MS6_A0927 was performed in a 50-µl reaction mixture containing 1 × Ex Taq buffer, 2 mM MgCl_2_, 0.2 mM dNTPs, each external primer at 1 µM (MS6_A0926F and MS6_A0928R) (Fig. 1), 1.25 U of Ex Taq DNA polymerase, and 100 ng of purified genomic DNA. Thermal cycling conditions were as follows: 95 °C for 4 min and 30 cycles of 95 °C for 30 s, 62 °C or 55 °C for 30 s, and 72 °C for 1.5 min. PCR products were purified with the NucleoSpin Gel and PCR Clean-up kit (Macherey*-*Nagel) or the QIAquick Gel Extraction kit (Qiagen, Valencia, CA, USA).

Samples positive for either *M or LH* were sequenced within these regions (Fig. 1). The PCR amplification for sequencing was performed in a 10-µl reaction mixture containing 1.5 µl of BigDye Terminator v.3.1 Ready Reaction Mix, 1.25 µl of 5 × BigDye Sequencing Buffer, each internal primer at 0.32 µM (lh*_*uni1–4 and m_uni1–4), and 40 ng of purified PCR product. The cycling condition was as follows: 96 °C for 1 min and 30 cycles of 96 °C for 10 s, 50 °C for 5 s, and 60 °C for 4 min. The reaction product was sequenced on the ABI 3130xl Genetic Analyzer platform (Applied Biosystems, Foster City, CA, USA). Sequence data determined in this study were submitted to DDBJ (DNA Data Bank of Japan, National Institute of Genetics, Mishima, Shizuoka, Japan) and published with the accession no. LC202659-LC202813.

For *M/LH* sequence profiling, DNA sequences of the targeted locus were aligned to one of two references: the 1,296-bp region of the MS6 strain (MS6_A0927) or the 1,600-bp region of the classical O1 strain O395 (from VC395_A0912 to VC395_A0913). The subclades were numbered by sequential assignment to each nucleotide sequence variation.

The sequence variations based on *M* and *LH* were compared with those from the nine SLST analyses. The sequence data of *kbl* (MS6_A0926) and *lysR* (MS6_A0928) and of the seven gene regions targeted by a multilocus sequence typing (MLST) procedure^21^ were extracted from the 186 genomes. Regions of sequences of the primer sets in each locus of the MLST were used. DNA sequences of each target gene were aligned to each reference sequence from MS6 (*kbl* and *lysR*) or N16961 (*adk*, *gyrB*, *mdh*, *metE*, *pntA*, *purM*, and *pyrC*). Arabic numbers were sequentially assigned to each sequence variation in each target region as described in Table 2. In addition, the number of alleles for MLST were determined based on the profile definitions in the *Vibrio cholerae* MLST databases (https://pubmlst.org/vcholerae/). The discrimination index was determined by calculation of the Simpson index of diversity, D^40^.

### Phylogenetic tree

Dendrograms were constructed by the neighbor-joining method using MEGA v.6.0^41^. Relationships among strains were assessed by genome-wide phylogenetic analysis. Coding sequences present as a single copy in genomes were analyzed using the Pan-genomes Analysis Pipeline v.1.02^26,42^.

## Electronic supplementary material

Supplementary Information
