# Supplementary material for: Vibrio cholerae embraces two major evolutionary traits as revealed by targeted gene sequencing
Source: Sci Rep. 2018 Jan 26;8:1631. doi: 10.1038/s41598-018-19995-7 (PMC5785995; doi:10.1038/s41598-018-19995-7)
Supplement: Supplementary file 1 — Supplementary Information [file 41598_2018_19995_MOESM1_ESM.pdf]

Vibrio cholerae embraces two major evolutionary traits as revealed by targeted gene sequencing

Kazuhisa Okada[1,2*], Warawan Wongboot[1], Siriporn Chantaroj[3], Wirongrong Natakuathung[1], Amonrattana Roobthaisong[1], Watcharaporn Kamjumphol[1], Fumito Maruyama[4], Taichiro Takemura[5], Ichiro Nakagawa[4], Makoto Ohnishi[6], Shigeyuki Hamada[1,2]

[1] Thailand-Japan Research Collaboration Center on Emerging and Re-emerging Infections (RCC-ERI), Nonthaburi, Thailand, [2]Research Institute for Microbial Diseases, Osaka University, Suita, Osaka, Japan, [3]National Institute of Health, Department of Medical Sciences, Ministry of Public Health, Nonthaburi, Thailand, [4] Department of Microbiology, Kyoto University, Sakyo-ku, Kyoto, Japan, [5]Vietnam Research Station, Institute of Tropical Medicine, Nagasaki University, Hanoi, Vietnam, [6] Department of Bacteriology, National Institute of Infectious Diseases , Shinjuku-ku, Tokyo, Japan.

[*]**Corresponding author**: Kazuhisa Okada

E-mail Address: kazuhisa@biken.osaka-u.ac.jp

Supplemental material

Table S1

Table S2

Figure S1

Figure S2

Figure S3

Figure S1. Dendrogram based on the *M* gene sequences of vibrios shows higher similarity within species than between species. Scale bars indicate nucleotide substitutions per site. *Vibrio cholerae* strains are highlighted in the light blue box.

Figure S2. Schematic representation of three types of gene arrangement found in the locus MS6_A0927 and hypothesized gene replacement of *M* and *LH* genes among *Vibrio cholerae* and *V. mimicus* strains. Gene arrangements among the eight strains grouped by the *M/LH* analysis are shown. Two *V. cholerae* strains N2_17 and 87395 showed a similar gene arrangement that contained both *M* (common M5) and *LH* (LH4 or LH19). Strain 87395 was phylogenetically most closely related with strain HE-09, which exhibited M5. The strains 87395, N56, N79, N80, and N83 exhibited identical LH4 and clustered in a deep branch of the dendrogram shown in Figure 2.

Figure S3. Multiplex PCR assay for rapid screening of six targeted genes including three virulence-related genes (*toxR, tcpA, ctxAB*), a seventh pandemic group-specific marker (*VC2346*), and *M/LH*. Lane M, 100-bp DNA ladder; lane 1, *V. cholerae* O1 El Tor strain N16961 (seventh cholera pandemic clone); lane 2, *V. cholerae* O1 classical strain 569B; lane 3, *V. cholerae* O1 El Tor strain MS6 (non-epidemic strain).

Table S1. Profiling of *Vibrio cholerae* and closely related species deposited in GenBank

Strain	Species*	Category	Year	Location	Serogroup	*ctxAB*	*tcpA*	*toxR*	*VC2346*	*LH*	*M*	Subclade	Accession no.
O395	VC	C	1965	India	O1	+	+	+	-	+	-	LH1	NC_012582.1/3.1
RC27	VC	U	1991	Indonesia	O1	+	+	+	-	+	-	LH1	ADAI01000000
V52	VC	C	1968	Sudan	O37	+	+	+	-	+	-	LH1	AAKJ00000000
95412	VC	C	1997	Mexco	O1	+	+	+	-	+	-	LH1	APFM00000000
NCTC 8457	VC	C	1910	Saudi Arabia	O1	-	+	+	-	+	-	LH2	AAWD00000000
M66-2	VC	C	1937	Indonesia	O1	-	+	+	-	+	-	LH2	CP001233/4
MAK757	VC	C	1937	Celebes Island	O1	+	+	+	-	+	-	LH2	AAUS_02000000
BX330286	VC	E	1986	Australia	O1	+	+	+	-	+	-	LH2	ACIA000000000
B33	VC	C	2004	Beira, Mozambique	O1	+	+	+	+	+	-	LH2	ACHZ01000000
MJ-1236	VC	C	1994	Matlab, Bangladesh	O1	+	+	+	+	+	-	LH2	NC_012667/8
RC9	VC	C	1985	Kenya	O1	+	+	+	+	+	-	LH2	ACHX00000000
N16961	VC	C	1975	Bangladesh	O1	+	+	+	+	+	-	LH2	NC_002505/6
2011EL-301	VC	E	2011	Taganrog, Russia	O1	+	+	+	+	+	-	LH2	AJFN00000000
CP1041(14)	VC	C	2004	Zambia	O1	+	+	+	+	+	-	LH2	ALDE00000000
CP1030(3)	VC	C	2008	Mexco	O1	+	+	+	+	+	-	LH2	ALCZ00000000
CP1032(5)	VC	C	1991	Mexico	O1	+	+	+	+	+	-	LH2	ALDA01000000
CP1033(6)	VC	C	2000	Mexico	O1	+	+	+	+	+	-	LH2	AJRL00000000
CP1038(11)	VC	C	2003	Zimbabwe	O1	+	+	+	+	+	-	LH2	ALDC00000000
CP1040(13)	VC	C	2004	Zambia	O1	+	+	+	+	+	-	LH2	ALDD00000000
CP1042(15)	VC	C	2010	Thailand	O1	+	+	+	+	+	-	LH2	ALDF00000000
CP1044(17)	VC	C	1991	Peru	O1	+	+	+	+	+	-	LH2	ALDG00000000
CP1047(20)	VC	C	1995	Peru	O1	+	+	+	+	+	-	LH2	ALDI00000000
CP1048(21)	VC	C	2010	Bangladesh	O1	+	+	+	+	+	-	LH2	ALDJ00000000
CP1050(23)	VC	C	2010	Bangladesh	O1	+	+	+	+	+	-	LH2	ALDK00000000
H1	VC	C	2010	Haiti	O1	+	+	+	+	+	-	LH2	AKGH00000000
HC-06A1	VC	C	2010	Haiti	O1	+	+	+	+	+	-	LH2	AGUI00000000
HC-17A1	VC	C	2010	Haiti	O1	+	+	+	+	+	-	LH2	AJRN00000000
HC-17A2	VC	C	2010	Haiti	O1	+	+	+	+	+	-	LH2	AJSI00000000
HC-19A1	VC	C	2010	Haiti	O1	+	+	+	+	+	-	LH2	AGUJ00000000
HC-20A2	VC	C	2010	Haiti	O1	+	+	+	+	+	-	LH2	ALDL00000000
HC-21A1	VC	C	2010	Haiti	O1	+	+	+	+	+	-	LH2	AGUK00000000
HC-23A1	VC	C	2010	Haiti	O1	+	+	+	+	+	-	LH2	AGUM00000000
HC-28A1	VC	C	2010	Haiti	O1	+	+	+	+	+	-	LH2	AGUN00000000
HC-32A1	VC	C	2010	Haiti	O1	+	+	+	+	+	-	LH2	AGUO00000000
HC-33A2	VC	C	2010	Haiti	O1	+	+	+	+	+	-	LH2	AGUP00000000
HC-37A1	VC	C	2010	Haiti	O1	+	+	+	+	+	-	LH2	AJSJ00000000
HC-38A1	VC	C	2010	Haiti	O1	+	+	+	+	+	-	LH2	AHGF00000000
HC_39A1	VC	C	2010	Haiti	O1	+	+	+	+	+	-	LH2	AFOV00000000
HC_40A1	VC	C	2010	Haiti	O1	+	+	+	+	+	-	LH2	ALDM00000000
HC_41A1	VC	C	2010	Haiti	O1	+	+	+	+	+	-	LH2	AFOK00000000
HC-42A1	VC	C	2010	Haiti	O1	+	+	+	+	+	-	LH2	ALDN00000000
HC-43A1	VC	C	2010	Haiti	O1	+	+	+	+	+	-	LH2	ALDO00000000
HC_46A1	VC	C	2010	Haiti	O1	+	+	+	+	+	-	LH2	AGUQ00000000
HC_47A1	VC	C	2010	Haiti	O1	+	+	+	+	+	-	LH2	ALDQ00000000
HC-48A1	VC	C	2010	Haiti	O1	+	+	+	+	+	-	LH2	ALDR00000000
HC-48B2	VC	C	2010	Haiti	O1	+	+	+	+	+	-	LH2	AFOL00000000
HC-49A2	VC	C	2010	Haiti	O1	+	+	+	+	+	-	LH2	AGUR00000000
HC-56A2	VC	C	2010	Haiti	O1	+	+	+	+	+	-	LH2	AFOM00000000
HC-57A2	VC	C	2010	Haiti	O1	+	+	+	+	+	-	LH2	ALDX00000000
HC-61A1	VC	C	2010	Haiti	O1	+	+	+	+	+	-	LH2	ALDZ00000000
HC-62A1	VC	C	2010	Haiti	O1	+	+	+	+	+	-	LH2	AGUS00000000
HC-62B1	VC	C	2010	Haiti	O1	+	+	+	+	+	-	LH2	AJRV00000000
HC-64A1	VC	C	2010	Haiti	O1	+	+	+	+	+	-	LH2	AJSO00000000
HC-65A1	VC	C	2010	Haiti	O1	+	+	+	+	+	-	LH2	AGUT00000000
HC-67A1	VC	C	2010	Haiti	O1	+	+	+	+	+	-	LH2	AGUU00000000
HC-68A1	VC	C	2010	Haiti	O1	+	+	+	+	+	-	LH2	AGUV00000000
HC-69A1	VC	C	2010	Haiti	O1	+	+	+	+	+	-	LH2	AGUW00000000
HC-71A1	VC	C	2010	Haiti	O1	+	+	+	+	+	-	LH2	AJSP00000000
HC-72A2	VC	C	2010	Haiti	O1	+	+	+	+	+	-	LH2	AGUX00000000
HC-77A1	VC	C	2010	Haiti	O1	+	+	+	+	+	-	LH2	AGUY00000000
HC-7A1	VC	C	2010	Haiti	O1	+	+	+	+	+	-	LH2	AJRW00000000
HC-80A1	VC	C	2010	Haiti	O1	+	+	+	+	+	-	LH2	AGVA00000000
HC-81A1	VC	C	2010	Haiti	O1	+	+	+	+	+	-	LH2	AGVB00000000
HC-81A2	VC	C	2010	Haiti	O1	+	+	+	+	+	-	LH2	AGVC00000000
HC-22A1	VC	C	2010	Haiti	O1	+	+	+	+	+	-	LH2	ALEA00000000
HC-70A1	VC	C	2010	Haiti	O1	+	+	+	+	+	-	LH2	AGUL00000000
HCUF01	VC	C	2010	Haiti	O1	+	+	+	+	+	-	LH2	AFON00000000
HFU_02	VC	C	2010	Haiti	O1	+	+	+	+	+	-	LH2	AFOO00000000
INDRE_91_1	VC	C	1991	Mexico	O1	+	+	+	+	+	-	LH2	AFOS00000000
116059	VC	C	1992	Brazil	O1	+	+	+	+	+	-	LH2	ADAK00000000
2009V-1046	VC	C	2009	USA	O1	+	+	+	+	+	-	LH2	APFJ00000000
2009V-1085	VC	C	2009	USA	O1	+	+	+	+	+	-	LH2	AHFX00000000
2009V-1096	VC	C	2009	USA	O1	+	+	+	+	+	-	LH2	AHFY00000000
2009V-1116	VC	C	2009	USA	O1	+	+	+	+	+	-	LH2	AHFZ00000000
2009V-1131	VC	C	2009	USA	O1	+	+	+	+	+	-	LH2	AHGA00000000
2010EL-1749	VC	C	2010	Cameroon	O1	+	+	+	+	+	-	LH2	AHGB00000000
2010EL-1786	VC	C	2010	Haiti	O1	+	+	+	+	+	-	LH2	AHGC00000000
2010EL-1792	VC	C	2010	Haiti	O1	+	+	+	+	+	-	LH2	NC_012667/8
2010EL-1798	VC	C	2010	Haiti	O1	+	+	+	+	+	-	LH2	AELJ00000000
2010EL-1961	VC	C	2010	Haiti	O1	+	+	+	+	+	-	LH2	AELI00000000
2010EL-2010H	VC	C	2010	Haiti	O1	+	+	+	+	+	-	LH2	AHGD00000000
2010EL-2010N	VC	C	2010	Haiti	O1	+	+	+	+	+	-	LH2	AHGE00000000
2010V-1014	VC	C	2010	USA	O1	+	+	+	+	+	-	LH2	AHGG00000000
2011EL-1089	VC	C	2010	Haiti	O1	+	+	+	+	+	-	LH2	AHGH00000000
2011EL-1133	VC	C	2011	Haiti	O1	+	+	+	+	+	-	LH2	AHGI00000000
2011EL-1137	VC	C	2009	South Africa	O1	+	+	+	+	+	-	LH2	AHGJ00000000
2011V-1021	VC	C	2011	Dominican Republic	O1	+	+	+	+	+	-	LH2	AHGK00000000
3500-05	VC	C	2005	USA	O1	+	+	+	+	+	-	LH2	AHGL00000000
3546-06	VC	C	2006	USA	O1	+	+	+	+	+	-	LH2	AHGM00000000
3554-08	VC	C	2008	USA	O1	+	+	+	+	+	-	LH2	AHGN00000000
3582-05	VC	C	2005	USA	O1	+	-	+	+	+	-	LH2	AHGP00000000
AG-7404	VC	C	1991	Bangladesh	O1	+	+	+	+	+	-	LH2	APFN00000000
AG-8040	VC	C	1991	Bangladesh	O1	+	+	+	+	+	-	LH2	APFO00000000

Strain	Species	Category	Year	Location	Serogroup	*ctxAB*	*tcpA*	*toxR*	*VC2346*	*LH*	*M*	Subclade	Accession no.
C6706	VC	C	1991	Peru	O1	+	+	+	+	+	-	LH2	AHGQ00000000
CIRS101	VC	C	2002	Dhaka, Bangladesh	O1	+	+	+	+	+	-	LH2	ACVW00000000
EC-0009	VC	E	2010	Bangladesh	O1	+	+	+	+	+	-	LH2	APFP01000000
EC-0012	VC	E	2010	Bangladesh	O1	+	+	+	+	+	-	LH2	APFQ00000000
EC-0027	VC	E	2011	Bangladesh	O1	+	+	+	+	+	-	LH2	APFR00000000
EC-0051	VC	E	2011	Bangladesh	O1	+	+	+	+	+	-	LH2	APFS00000000
EDC-020	VC	E	2010	Bangladesh	O1	+	+	+	+	+	-	LH2	APFT00000000
EDC-022	VC	E	2010	Bangladesh	O1	+	+	+	+	+	-	LH2	APFU00000000
EM-1536	VC	E	2010	Bangladesh	O1	+	+	+	+	+	-	LH2	APFV00000000
EM-1546	VC	E	2010	Bangladesh	O1	+	+	+	+	+	-	LH2	APFW00000000
EM-1626	VC	E	2010	Bangladesh	O1	+	+	+	+	+	-	LH2	APFX00000000
EM-1727	VC	E	2011	Bangladesh	O1	+	+	+	+	+	-	LH2	APFZ01000000
G4222	VC	C	2001	South Africa	O1	+	+	+	+	+	-	LH2	ANNB00000000
Nep-21106	VC	C	2003	Nepal	O1	+	+	+	+	+	-	LH2	APGE00000000
NHCC_004A	VC	C	2010	Bangladesh	O1	+	+	+	+	+	-	LH2	APGA01000000
NHCC-006C	VC	C	2010	Bangladesh	O1	+	+	+	+	+	-	LH2	APGB00000000
NHCC-010F	VC	C	2010	Bangladesh	O1	+	+	+	+	+	-	LH2	APGD00000000
Nep-21113	VC	C	2003	Nepal	O1	+	+	+	+	+	-	LH2	APGF00000000
VC1761	VC	C	2009	Malaysia	O1	+	+	+	+	+	-	LH2	AMBS00000000
MO10	VC	C	1992	Madras, India	O139	+	+	+	+	+	-	LH2	AAKF03000000
4260B	VC	C	1993	Bangladesh	O139	+	+	+	+	+	-	LH2	AMVL00000000
VC4370	VC	E	2008	Selango, Malaysia	O139	+	+	+	+	+	-	LH2	AMBT00000000
MS6	VC	C	2008	Thailand	O1	+	+	+	-	-	+	M1	AP014524/5
P-18785	VC	C	2005	Russia	O1	-	+	+	-	-	+	M1	ANHS00000000
2740-80	VC	E	1980	US Gulf coast, USA	O1	-	+	+	-	-	+	M2	AAUT01000000
3569-08	VC	E	2008	US Gulf coast, USA	O1	+	+	+	-	-	+	M2	AHGO00000000
CP1110	VC	C	2010	Florida, USA	O75	+	+	+	-	-	+	M3	AMWF00000000
CP1111	VC	C	2010	Florida, USA	O75	+	+	+	-	-	+	M3	AMWS00000000
CP1112	VC	C	2010	Florida, USA	O75	+	+	+	-	-	+	M3	AMWT00000000
CP1113	VC	C	2010	Florida, USA	O75	+	+	+	-	-	+	M3	AMWU00000000
CP1114	VC	C	2010	Florida, USA	O75	+	+	+	-	-	+	M3	AMWV00000000
CP1115	VC	C	2010	Florida, USA	O75	+	+	+	-	-	+	M3	AMWR00000000
CP1116	VC	C	2010	Florida, USA	O75	+	+	+	-	-	+	M3	ANNM00000000
CP1117	VC	C	2010	Florida, USA	O75	+	+	+	-	-	+	M3	AMWW00000000
HC-41B1	VC	C	2010	Haiti	NAG	-	-	+	-	-	+	M4	AJRP00000000
HC-43B1	VC	C	2010	Haiti	NAG	-	-	+	-	-	+	M4	ALDP00000000
HC-44C1	VC	C	2010	Haiti	NAG	-	-	+	-	-	+	M4	AJSK00000000
HC_46B1	VC	C	2010	Haiti	NAG	-	-	+	-	-	+	M4	AJSL00000000
116063	VC	C	1978	Brazil	O1	-	-	+	-	-	+	M4	APFK00000000
TM11079-80	VC	E	1980	Brazil	O1	-	-	+	-	-	+	M4	ACHW00000000
Amazonia	VC	C	1991	Brazil	O1	-	-	+	-	-	+	M4	AFSV00000000
HE-09	VC	E	2010	Haiti	NAG	-	-	+	-	-	+	M5	AFOP00000000
87395	VC	C	1983	Mexico	O1	-	-	+	-	+	+	LH4M5	APFL00000000
HC-02C1	VC	C	2010	Haiti	NAG	-	-	+	-	-	+	M6	AJSH00000000
HC-1A2	VC	C	2010	Haiti	NAG	-	-	+	-	-	+	M6	AJRO00000000
HC-50A1	VC	C	2010	Haiti	NAG	-	-	+	-	-	+	M6	ALDS00000000
HC-51A1	VC	C	2010	Haiti	NAG	-	-	+	-	-	+	M6	ALDT00000000
HC-52A1	VC	C	2010	Haiti	NAG	-	-	+	-	-	+	M6	ALDU00000000
HC-55A1	VC	C	2010	Haiti	NAG	-	-	+	-	-	+	M6	ALDV00000000
HC-55B2	VC	C	2010	Haiti	NAG	-	-	+	-	-	+	M6	AJSM00000000
HC-55C2	VC	C	2010	Haiti	NAG	-	-	+	-	-	+	M6	AJRR00000000
HC-56A1	VC	C	2010	Haiti	NAG	-	-	+	-	-	+	M6	ALDW00000000
HC-57A1	VC	C	2010	Haiti	NAG	-	-	+	-	-	+	M6	ALDY00000000
HC-59A1	VC	C	2010	Haiti	NAG	-	-	+	-	-	+	M6	AJRS01000000
HC-59B1	VC	C	2010	Haiti	NAG	-	-	+	-	-	+	M6	AJSN00000000
HC-60A1	VC	C	2010	Haiti	NAG	-	-	+	-	-	+	M6	AJRT00000000
HC-61A2	VC	C	2010	Haiti	NAG	-	-	+	-	-	+	M6	AJRU00000000
HC-78A1	VC	C	2010	Haiti	NAG	-	-	+	-	-	+	M6	AGUZ00000000
HC-02A1	VC	C	2010	Haiti	NAG	-	-	+	-	-	+	M6	AFOT00000000
HE-40	VC	E	2010	Haiti	NAG	-	-	+	-	-	+	M7	AJRX00000000
HE-46	VC	E	2010	Haiti	NAG	-	-	+	-	-	+	M7	AJRY00000000
HE39	VC	E	2010	Haiti	NAG	-	-	+	-	-	+	M7	AFOQ00000000
SX_4	VM	C	2009	China	U	+	+	+	-	-	+	M8	ADOO00000000
VM573	VM	C	1990s	USA	U	+	+	+	-	-	+	M8	ACYV00000000
CP1046(19)	VC	C	1995	Peru	O1	-	+	+	+	+	-	LH3	ALDH00000000
AM-19226	VC	C	2001	Bangladesh	O39	-	-	+	-	-	+	M9	AATY01000000
HE-25	VC	E	2010	Haiti	NAG	-	-	+	-	-	+	M10	ALEC00000000
12129(1)	VC	E	1985	Australia	O1	-	-	+	-	+	-	LH5	ACFQ00000000
EM-1676A	VC	E	2011	Bangladesh	O1	-	-	+	-	+	-	LH6	APFY00000000
1587	VC	C	1994	Lima, Peru	O12	-	-	+	-	+	-	LH7	AAUR01000000
MZO-2	VC	C	2001	Bangladesh	O14	-	-	+	-	+	-	LH8	AAWF01000000
MZO-3	VC	C	2001	Bangladesh	O37	-	-	+	-	+	-	LH9	AAUU00000000
RC385	VC	E	1998	MD, USA	O135	-	-	+	-	+	-	LH10	AAKH03000000
BJG-01	VC	C	2010	Mississippi, USA	NAG	-	-	+	-	+	-	LH11	AFOU00000000
HE-16	VC	E	2010	Haiti	NAG	-	-	+	-	+	-	LH12	ALEB00000000
PS15	VC	E	U	WA, USA	O106	-	-	+	-	+	-	LH13	AIJR00000000
VL426	VC	E	U	Maidstone, Kent, UK	NAG	-	-	+	-	+	-	LH14	ACHV00000000
CP1035(8)	VC	C	2004	Mexico	U	-	-	+	-	-	+	M11	AJRM00000000
NHCC-008D	VC	C	2010	Bangladesh	O1	-	-	+	-	-	+	M12	APGC00000000
VC35	VC	C	2004	Malaysia	O1	-	-	+	-	-	+	M13	AMBR00000000
V51	VC	C	1987	USA	O141	+	+	+	-	-	+	M14	AAKI020000000
HE-45	VC	E	2010	Haiti	NAG	-	-	+	-	-	+	M15	ALED00000000
CP1037(10)	VC	C	2003	Mexico	U	-	+	+	-	-	+	M16	ALDB00000000
623-39	VC	E	2002	Bangladesh	NAG	-	-	+	-	-	+	M17	AAWG00000000
TMA21	VC	E	1982	Brazil	NAG	-	-	+	-	-	+	M18	ACHY00000000
CT 5369-93	VC	E	1993	Brazil	O1	-	-	+	-	+	-	LH15	ADAL00000000
HE48	VC	E	2010	Centre, Haiti	NAG	-	-	+	-	-	+	M19	AFOR00000000
CAIM602	VM	C	1981	North Carolina, USA	U	-	-	+	-	-	+	M20	AOMO00000000
MB-451	VM	C	U	Bangladesh	U	-	-	+	-	-	+	M21	ADAF00000000
VM223	VM	E	U	Sao Paulo, Brazil	U	-	-	+	-	-	+	M22	ADAJ00000000
VM603	VM	U	1990s	Brazil	U	-	-	+	-	-	+	M23	ACYU00000000
RC341	VMe	E	1998	MD, USA	O153	-	-	-	-	-	+	M24	ACZT00000000
RC586	VPa	E	1999	MD, USA	O133	-	-	-	-	-	+	M25	ADBD00000000

Abbreviations: C, human isolate; E, environmental isolate; NAG, non-O1/ non-O139 *V. cholerae; U, unknown;* VC*, Vibrio cholerae;* VM, *V. mimicus;* VMe, *V. metecus;* VPa*, V. parilis.*

Table S2. Primers for multiplex PCR assay and targeted gene sequencing of the MS6_A0927

Target gene	Primer name	Nucleotide sequence (5'→3')	Amplicon size (bp)	Reference
Multiplex PCR				
M	metY-F	GCGTGAAACCGGAGATGATCC	353	(1)
	lysR-R	AGCGCAGAAGGTGTTACGCCA		(1)
LH	luxR-F	TAGCTCACCGCGAGCTCGTTG	521	(1)
	lysR-R	AGCGCAGAAGGTGTTACGCCA		(1)
VC2346	VC2346-F	CAACTGTGCTAGCAGTTGCC	243	This study
	VC2346-R	TCAGAGTCATAGATTGAAGC		This study
ctxA-B	ctxA-B-F	GTGTGTTGTGGTATTCTGCAC	702	This study
	ctxA-B-R	TCATGCAAGAGGAACTCAGAC		This study
tcpA	tcpA-F	ACCGGTCAAGAGGGTATGACA	110	This study
	tcpA-R	CTGCGAATCAATCGCACGCTG		This study
toxR	toxR-F	TGCGTAAGGTTATGTTTTCCC	844	This study
	toxR-R	GTTCGGATTAGGACACAACTC		This study
Targeted gene sequencing				
MS6_A0927	MS6_A0926F	CGGATTTGCTGATAAAAAGCAGAAGA	c.a. 2,130 or	This study
	MS6_A0928R	GCGGTAAAACTGCCTTCATTRACCAC	1,960 or over	This study
Sequence primer				
M	m_uni1-R	TCAATCCCTTGGCTTGGC		This study
	m_uni2-R	AGGATCATGAAGGCGTTCA		This study
	m_uni3-F	TCAACTACGCGATTCTGAC		This study
	m_uni4-F	GTACACCGAAGCCTTTGG		This study
LH	lh_uni1-R	ACCTCTTGGCTGTCAGGC		This study
	lh_uni2-F	ATCTTCTTTCAGGCAGCC		This study
	lh_uni3-F	GATAGTCCGCTTGCAGGC		This study
	lh_uni4-F	ATATTGCCCTATAACTCG		This study

Supplemental reference

(1) Okada, K., Na-Ubol, M., Natakuathung, W., Roobthaisong, A., Maruyama, F., Nakagawa, I., Chantaroj, S., and Hamada, S. (2014). Comparative genomic characterization of a Thailand-Myanmar isolate, MS6, of *Vibrio cholerae* O1 El Tor, which is phylogenetically related to a "US Gulf Coast" clone. *PLoS One* 9**,** e98120.

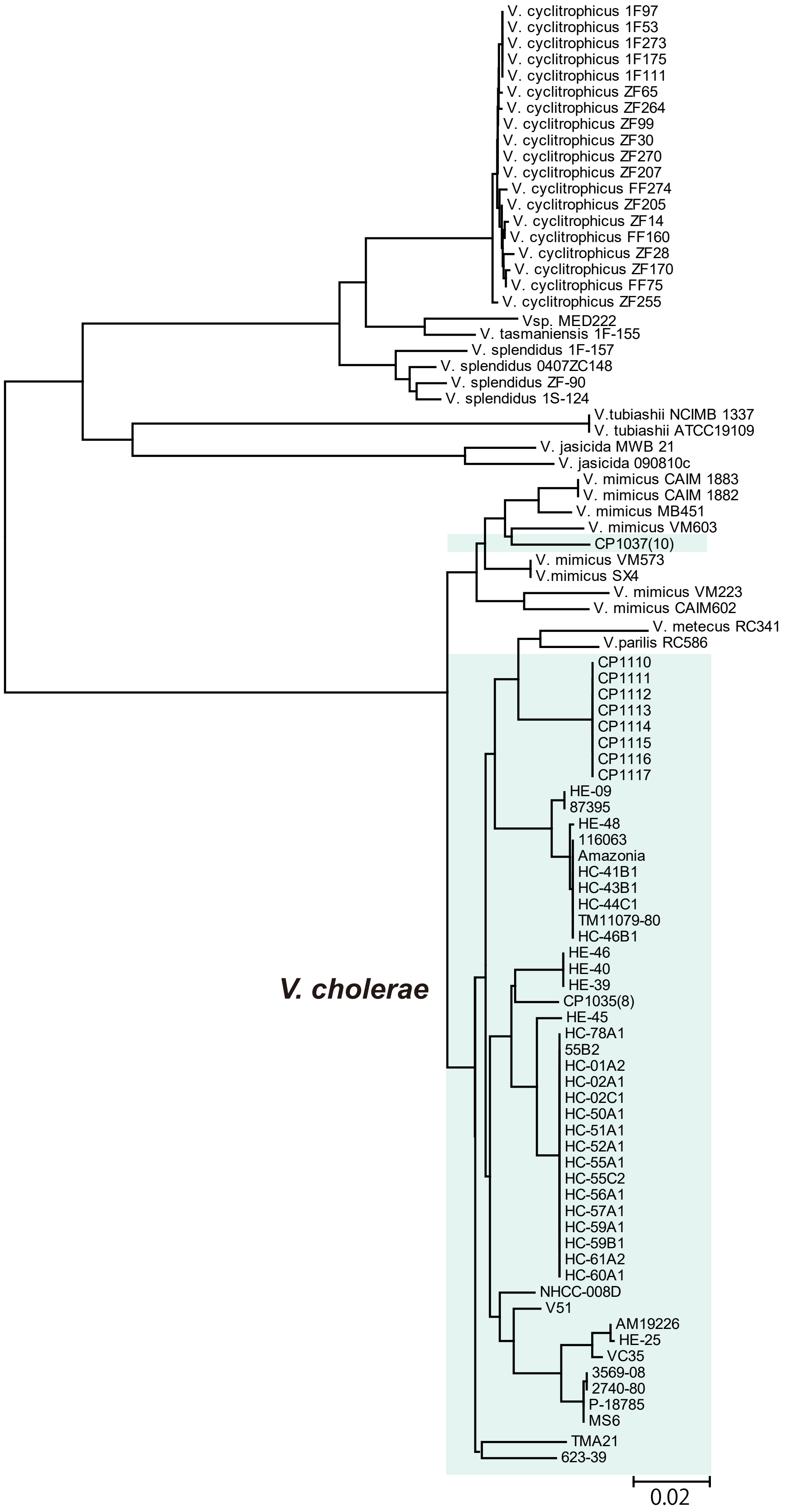

Figure S1. Dendrogram based on the *M* gene sequences of vibrios shows higher similarity within species than between species. Scale bars indicate nucleotide substitutions per site. *Vibrio cholerae* strains are highlighted in the light blue box.

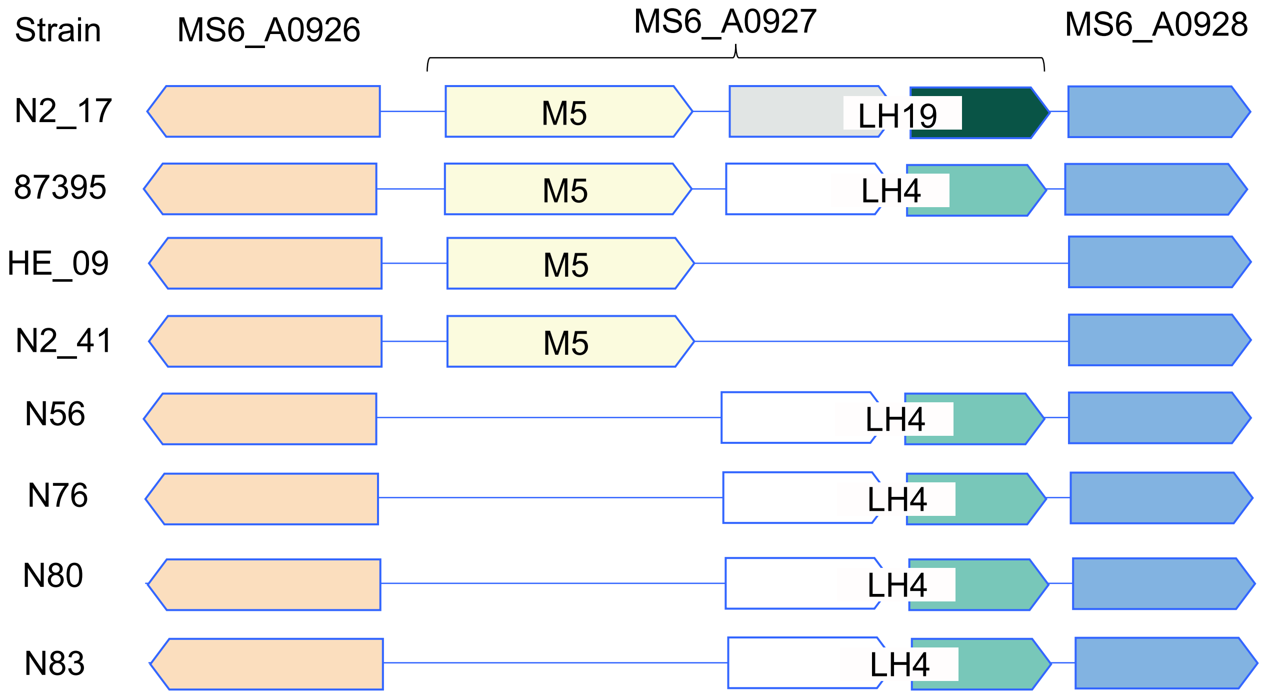

Figure S2. Schematic representation of three types of gene arrangement found in the locus MS6_A0927 and hypothesized gene replacement of *M* and *LH* genes among *Vibrio cholerae* and *V. mimicus* strains. Gene arrangements among the eight strains grouped by the *M/LH* analysis are shown. Two *V. cholerae* strains N2_17 and 87395 showed a similar gene arrangement that contained both *M* (common M5) and *LH* (LH4 or LH19). Strain 87395 was phylogenetically most closely related with strain HE-09, which exhibited M5. The strains 87395, N56, N79, N80, and N83 exhibited identical LH4 and clustered in a deep branch of the dendrogram shown in Figure 2.

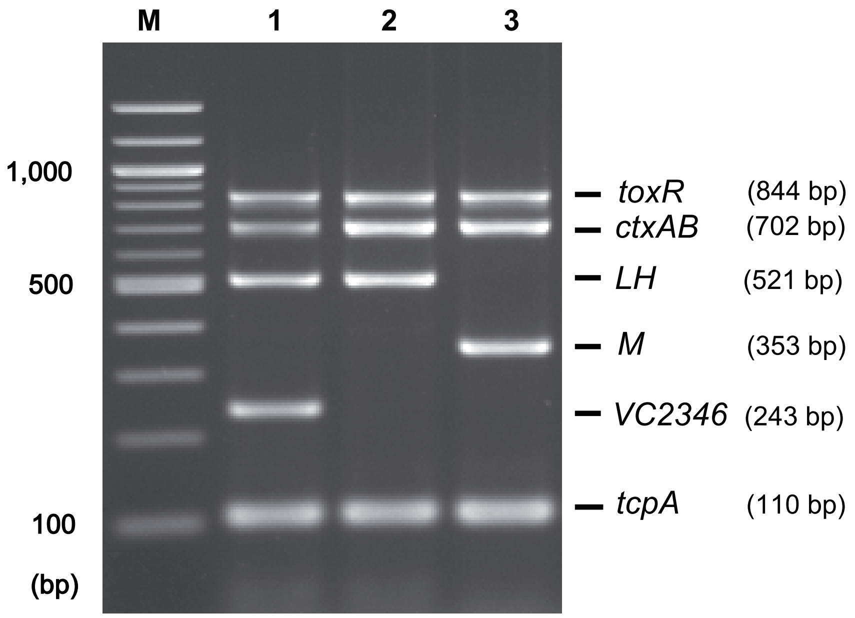

Figure S3. Multiplex PCR assay for rapid screening of six targeted genes including three virulence-related genes (*toxR, tcpA, ctxAB*), a seventh pandemic group-specific marker (*VC2346*), and *M/LH*. Lane M, 100-bp DNA ladder; lane 1, *V. cholerae* O1 El Tor strain N16961 (seventh cholera pandemic clone); lane 2, *V. cholerae* O1 classical strain 569B; lane 3, *V. cholerae* O1 El Tor strain MS6 (non-epidemic strain).
